# Incision of the Tunica Vaginalis and Albuginea Testes for Relief of Testitis Occurring as a Sequel of Parotitis

**Published:** 1881-07

**Authors:** Thos. H. Carroll

**Affiliations:** Smithport, Pa.


### INCISION OF THE TUNICA VAGINALIS AND ALBUGINEA TESTES FOR RELIEF OF TESTITIS OCCURRING AS A SEQUEL OF PAROTITIS.

*Editors Buffalo Medical and Surgical Journal:*

An epidemic of "Mumps" prevailing in this locality during the last four months, was remarkable for severity of symptoms, the disease frequently setting in with chills, fever and occasionally delirium. But the most remarkable feature of the disorder was tendency to metastasis to the testis, seven cases occurring in my own practice; one case especially severe, the history of which may not prove uninteresting to your readers.

A. H., aged 38, contracted the disease in a rather mild form, both parotid glands participated, fever slight during the first four days; the patient sat up and was quite comfortable until the morning of the fifth day, when he experienced a painful feeling in the right testicle. Examination revealed tenderness, superficial œdemas and slight engorgement. The same evening the patient suddenly became—as he expressed it—"sick to his stomach," was taken with a chill and acceleration of temperature. The usual remedies were resorted, to depletion by saline cathartics, hot fomentations, etc., without avail. On the ninth day of the disease the testicle felt like a billiard-ball and was extremely painful. Dreading suppuration, I resolved to relieve the tension. With a small curved bistoury I invaded the scrotum, anteriorly and opposite the central anterior aspect of the affected testicle, and carefully divided the tense tunica vaginalis and albuginea. Relief was instantaneous and permanent, entire recovery occurring within a week. A slight tenderness of testicle resulted from active exercise for a week or two, when it entirely disappeared.

Prof. Flint in his work on "Practice" states that "Metastasis is of rare occurrence" and adds, "I have seen but one case." I am disposed to consider the numerous cases occurring in my own practice, good evidence either, of an unusually severe epidemic of "Mumps," or incorrectness of Professor Flint's observations. Faithfully Yours,

Thos. H. Carroll, M. D.,

June 10th, 1881. Smithport, Pa.

